# Cerebral blood flow decrease as an early pathological mechanism in Alzheimer's disease

**DOI:** 10.1007/s00401-020-02215-w

**Published:** 2020-08-31

**Authors:** Nils Korte, Ross Nortley, David Attwell

**Affiliations:** Department of Neuroscience, Physiology and Pharmacology, University College London, Gower Street, London, WC1E 6BT UK

**Keywords:** Alzheimer’s, Cerebral blood flow, Capillary, Amyloid β, Pericyte, Neutrophil

## Abstract

Therapies targeting late events in Alzheimer’s disease (AD), including aggregation of amyloid beta (Aβ) and hyperphosphorylated tau, have largely failed, probably because they are given after significant neuronal damage has occurred. Biomarkers suggest that the earliest event in AD is a decrease of cerebral blood flow (CBF). This is caused by constriction of capillaries by contractile pericytes, probably evoked by oligomeric Aβ. CBF is also reduced by neutrophil trapping in capillaries and clot formation, perhaps secondary to the capillary constriction. The fall in CBF potentiates neurodegeneration by upregulating the BACE1 enzyme that makes Aβ and by promoting tau hyperphosphorylation. Surprisingly, therefore, CBF reduction may play a crucial role in driving cognitive decline by initiating the amyloid cascade itself, or being caused by and amplifying Aβ production. Here, we review developments in this area that are neglected in current approaches to AD, with the aim of promoting novel mechanism-based therapeutic approaches.

## Introduction

Thirty years of research have given us a broad understanding of many mechanisms contributing to Alzheimer’s disease [99], but over 400 clinical trials of drugs targeting these pathways have largely failed to reduce cognitive decline [47, 109, 136]. Identification of the amyloid β protein (Aβ) as the major component of amyloid plaques, together with genetic evidence, initially indicated that dysfunction of the processing of amyloid precursor protein (APP) was the cause of Aβ plaque deposition and downstream tau tangle formation and neuronal dysfunction [59]. Subsequent work led to the conclusion that the level of soluble Aβ oligomers, and of hyperphosphorylation of the cytoskeletal protein tau that is induced by Aβ [62, 91], correlated better with cognitive decline than did plaque level [7, 57, 89, 123].

**Fig. 1:**
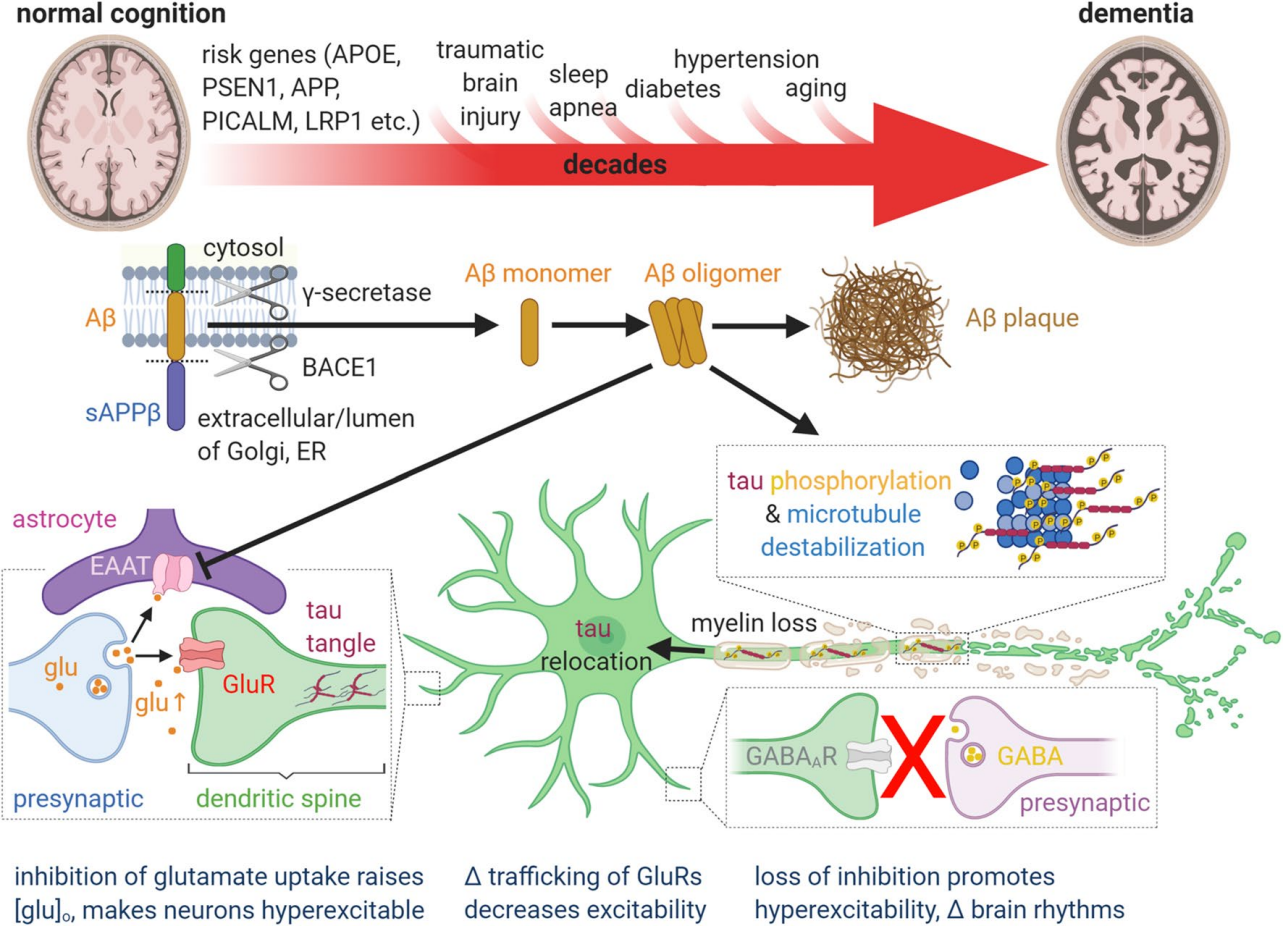
Current, generally held ideas about the pathology underlying Alzheimer’s disease (see main text for details). The transition from normal cognition to dementia, over decades, is promoted by the risk factors shown above the large red arrow. Aβ is produced from amyloid precursor protein (APP) by the action of the γ secretase and β secretase (BACE1) as monomers, but these can then form soluble oligomers, which ultimately form extracellular precipitates as amyloid plaques. Aβ oligomers inhibit astrocyte glutamate uptake (EAAT), thus potentiating the action of synaptically released glutamate (glu). This, together with a loss of GABAergic inhibition, leads to some neurons becoming hyperexcitable. Meanwhile, Aβ oligomers also induce hyperphosphorylation of axonal microtubule-associated tau, which leads to tau redistributing partly to dendrites where it disrupts trafficking of glutamate receptors and thus depresses excitation and neuronal firing. These synaptic effects, and Aβ- and/or tau-induced loss of axonal myelin, may induce cognitive dysfunction well before synapses are lost and neurons die. The levels of Aβ oligomers and hyperphosphorylated tau correlate better with cognitive decline than does the level of Aβ plaques

There are established mechanisms by which Aβ oligomers and hyperphosphorylated tau can contribute to neuronal dysfunction and cognitive decline before synaptic and neuronal damage, and even before Aβ plaque and tau tangle deposition (Fig. 1). Aβ oligomers reduce glutamate uptake [92, 94, 199]. This raises the extracellular glutamate level and increases neuronal excitability [19, 20], which alters synaptic plasticity [92, 94] and in extremis may induce excitotoxicity [60]. Tau phosphorylation leads to soluble tau relocating from axonal microtubules into dendritic spines, where it alters postsynaptic glutamate receptor trafficking or anchoring (of both AMPA and NMDA receptors) and thus suppresses excitatory postsynaptic currents and neuronal activity [21, 67]. These changes may be particularly important when they affect the function of interneurons, which play a key role in generating oscillatory activity that contributes to cognitive function [63, 70, 176].

Preclinical AD has therefore been conceptualised as a synaptic disease [157] driven by Aβ and downstream tau phosphorylation, with loss of synapses and cells occurring late in the disorder. However, individuals can be cognitively normal while having plaque levels as high as those in Alzheimer’s dementia patients, and the same is true for levels of soluble Aβ oligomers [39]. This could reflect the presence of compensating protective mutations or developmental differences in the subjects with high Aβ levels. Alternatively, together with the fact that attempts to prevent cognitive decline—by blocking Aβ production, removing Aβ with antibodies or preventing tau phosphorylation—have all failed clinically (with one possible exception [68]), these data may suggest that there is some other variable that is missing from our understanding of the Aβ-tau cascade. Previously it has been suggested that the vasculature might provide such a factor, in the form of hypertension, impaired blood–brain barrier function, decreased Aβ clearance to the blood, vascular oxidative stress and inflammatory damage, or reduced neurovascular coupling at the arteriolar level [71, 198]. In this review, we show that new evidence reveals that a major missing variable is cerebral blood flow—and specifically its control by capillary pericytes.

## Large decreases of cerebral blood flow occur early in AD

Cerebral blood flow and glucose metabolism are reduced, and the brain’s vascular resistance is increased, in human AD [17, 107, 112, 115, 144, 151, 163, 165, 188] and in mice overexpressing amyloid precursor protein (APP) to mimic AD [129]. This also occurs in humans and mice expressing the ApoE4 protein, which predisposes towards AD [111, 148, 162, 163, 172]. The CBF reduction reaches over 50% in some brain areas [5], which is expected to reduce the activity of the Na/K pump (the main consumer of ATP in the CNS: [8]) and all processes dependent on it (including maintenance of the resting potential and glutamate uptake). It will also lead to adenosine generation, which is known to suppress glutamate release [43], and will produce numerous cell biological changes including changes of the balance of protein synthesis and degradation [173].

Although these changes could simply reflect tissue atrophy in AD [30], with a corresponding loss of blood supply and metabolism, they are associated with hypoxia [114] and it has been reported that the decrease of metabolism is greater than would be expected for the amount of atrophy occurring [165]. Furthermore, the observations of focal constrictions in capillaries from human AD brains [83], constriction of capillaries near plaques in human AD brains [58], and reduced neurovascular coupling and cerebrovascular reactivity in AD mice [48, 174] suggest that blood flow may be reduced by decreases in vessel diameter, and not just by loss of blood vessels.

Chronic blood flow reductions of 50% are expected to cause significant cognitive changes: a sustained reduction in CBF beyond 20% in humans leads to loss of ability to sustain attention, while a reduction beyond 30% in rats impairs spatial memory [105, 177]. A causal influence of blood flow changes on the cognitive changes at the onset of Alzheimer’s disease, before synapses or neurons are lost, is suggested by the fact that the reduction of cerebral blood flow starts early in preclinical AD [107, 180], with a faster onset than the deposition of Aβ or tau [76], and the fall of metabolism is also an early event [81, 115]. Furthermore, these changes correlate with cognitive decline [17, 112, 151].

## Cerebral blood flow decreases in AD largely reflect pericytes constricting capillaries

**Fig. 2:**
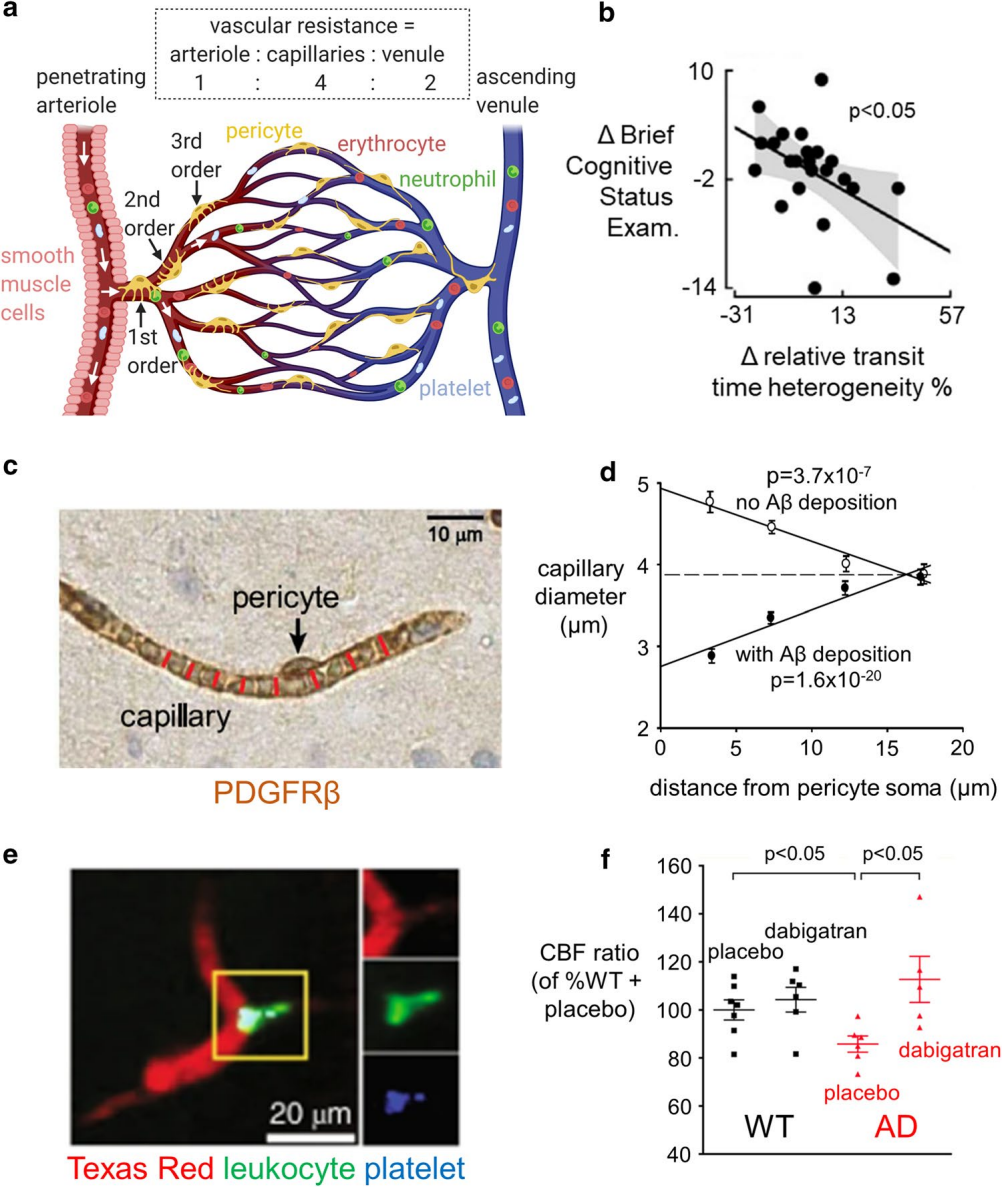
The role of pericytes in the physiology and Alzheimer’s-related pathology of the brain circulation. **a** Schematic diagram of the vascular bed (colour of blood represents oxygenation), indicating the relative resistance in the capillaries compared to penetrating arterioles and venules, for flow from the pial surface down an arteriole to layer 4, through the capillary bed, and returning to the pial surface through a venule [49]. Capillary diameter can be adjusted by a population of pericytes (yellow) that are contractile, which are located on at least the first four branch orders (see labels) of the capillary bed [56]. Blood flowing through capillaries with pericytes that are contracting to reduce the diameter will flow more slowly and so has a longer capillary transit time than blood flowing through capillaries with relaxed pericytes, thus generating capillary transit time heterogeneity (CTTH). **b** In patients with AD, CTTH (shown as a % change) increases as cognitive power (assessed with the Brief Cognitive Status Examination) declines (from Fig. 5A of [128], reproduced courtesy of John Wiley and Sons). **c**, **d** Capillary imaged in right frontal cortex biopsy from a dementing patient lacking Aβ deposition (**c**) and plot of mean capillary diameter versus distance from pericyte somata (**d**) in similar patients lacking or showing Aβ deposition (from Fig. 4A, D of [133]). Patients depositing Aβ show a large constriction near the pericyte somata. **e** Neutrophil (green) occluding a branch (to the right) of a capillary in AD mouse cortex (from Fig. 2A of [26], reproduced courtesy of Springer Nature). **f** Reducing clotting with dabigatran in WT and AD mice (from Fig. 3B of [25], reproduced courtesy of Elsevier Press) increases CBF in AD mice

The brain is unusual in that most of the resistance in its vascular bed is in capillaries (Fig. 2a) rather than in arterioles or venules [49], and cerebral blood flow is controlled not only by vascular smooth muscle cells wrapped around arterioles, but also by contractile pericytes which enwrap at least the first 4 branch orders of capillaries from the penetrating arteriole [9, 56, 82, 84, 143, 152, 187]. Contraction of these pericytes produces localised capillary constrictions near the pericyte somata (where most of the circumferential processes of the pericytes are located [133]) and could account for the focal capillary constrictions seen anatomically in capillaries isolated from human AD brains [83].

Despite the award of the Nobel Prize to Krogh [87] for his discovery of contractile elements on capillaries which act independently of smooth muscle cells on arterioles, there has been some controversy in the literature about whether pericytes are in fact contractile. However, this debate has now largely been resolved. The Zlokovic group [127] assessed in vitro, ex vivo and in vivo studies on pericyte contractility and found that 37 out of 39 separate papers reported that pericytes display contractility (and one of the 2 remaining papers [65] actually showed pericytes contracting, but renamed these cells smooth muscle cells: see [9] for discussion). Furthermore, whereas contractility had previously been demonstrated most clearly for pericytes on the 1st–4th branch orders of capillary measured from a penetrating arteriole [56, 65] which express the highest levels of α-smooth muscle actin, innovations in histochemistry have revealed that even higher branch order pericytes express this contractile protein [3] and optogenetic experiments have shown that these higher branch order pericytes can also regulate capillary diameter and blood flow [www.biorxiv.org/content/10.1101/2020.03.26.008763v1].

Functional indications that capillary pericyte-mediated control of CBF is disrupted in AD have been provided by measurements of the capillary transit time of the blood, and its heterogeneity. Magnetic resonance imaging (MRI) experiments on humans and optical imaging experiments on AD mice have found that AD leads to both a prolongation of the capillary transit time and an increase in its heterogeneity, as if some capillary pericytes became more constricted than others [38, 54]. Furthermore, in humans, these changes correlate with cognitive decline (Fig. 2b), as measured by the Brief Cognitive Status Examination [128].

By analysing images of brain biopsies of patients who consulted neurologists for dementia of unknown cause (Fig. 2c), Nortley et al. [133] demonstrated that patients developing AD have capillary blood flow restricted as a result of capillary constriction. This was shown to be due to pericytes by examining how capillary diameter varied as a function of the distance along the capillary from the pericyte soma (Fig. 2d). Patients depositing Aβ and tau tangles showed a constriction at the pericyte soma relative to positions between pericytes on the capillary. This increased rapidly with the amount of Aβ deposited, suggesting a CBF reduction mechanism that occurs early in the development of the disease (before accumulation of Aβ in and around vascular cells—cerebral amyloid angiopathy—leads to pericyte loss), as is also seen in live imaging of CBF in AD patients [107]. In contrast, in patients lacking Aβ and tau deposition, capillaries showed a larger diameter near the pericyte soma, perhaps because pericytes normally induce growth of the endothelial tube. The difference in the spatial profile of capillary diameter between AD and non-AD patients was estimated to be able to generate a reduction in CBF of ~ 50%, similar to that found in AD patients in vivo [5].

In AD mouse models, live cortex imaging through a cranial window, or reconstructing the hippocampal vasculature of fixed brains, also showed a reduction of mean capillary diameter compared to normal mice [55, 133, 193], which in cortex reflected capillary constriction near pericyte somata [133]. Nortley et al. [133] further demonstrated that, in the AD model mouse they used, neither arterioles nor venules had an altered diameter, implying that the reduction of CBF is generated by capillaries (although this still remains to be shown for human AD and other AD mouse models).

## Mechanism of CBF decrease

**Fig. 3:**
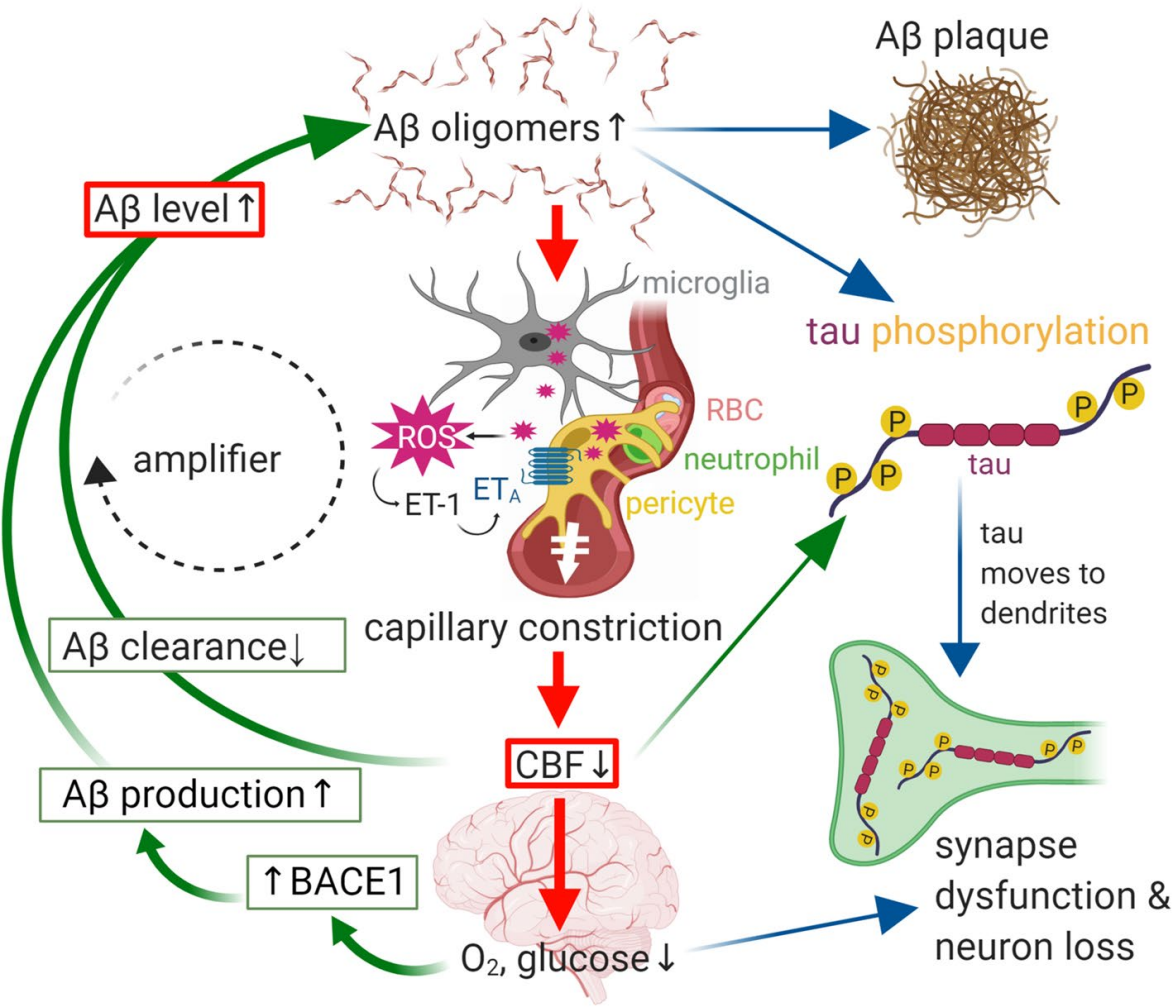
Schematic diagram showing how the amyloid beta and tau cascades can be initiated from two entry points (red boxes): (i) a decrease of cerebral blood flow (CBF) which lowers brain O_2_ and glucose and thus upregulates the enzyme (BACE1) that makes Aβ or (ii) an increase in Aβ level due to more production or less clearance of Aβ. Aβ oligomers can aggregate into plaques, but also evoke ROS production from microglia and pericytes, which triggers the release of endothelin-1 (ET-1) from a yet-to-be-determined cell type [133]. Activation of ET_A_ receptors on pericytes leads to capillary constriction and a decrease of CBF, lowering the levels of O_2_ and glucose. Both a rise of Aβ oligomer concentration and a fall of blood flow lead to hyperphosphorylation of tau, which relocates from axonal microtubules to dendrites, causing synapse dysfunction. Together with myelin loss this leads to cognitive decline. The fall of CBF will also contribute to impaired cognition

Although the mechanism of the long-term pericyte-mediated constriction of capillaries that occurs in human AD brains has not yet been definitively identified, short-term application of Aβ oligomers (both Aβ_1–42_ and Aβ_1–40_, at nanomolar concentrations similar to those present in AD) to human or rodent brain slices evoked capillary constriction [133] mediated by reactive oxygen species (ROS) generation and activation of endothelin A (ET_A_) receptors (Fig. 3). It is plausible that this signalling pathway is also responsible for capillary constriction in the human AD brain, since the concentrations of both ROS and endothelin-1 are known to be elevated in human AD [10, 114, 135]. The locus of ROS generation is debated, with Park et al. [141] suggesting it to be perivascular macrophages, while Nortley et al. [133] found that ROS are generated by microglia and pericytes. ET_A_ receptors are known to be expressed on all classes of pericyte [190] and their activation in AD is consistent with the elevated level of extracellular endothelin-1 (ET) found in post-mortem AD brains [113, 135].

Release of inflammatory mediators generated during AD may also contribute to the decrease of CBF occurring. Interleukin-1β is generated when microglial and astrocyte inflammasomes are activated by oligomeric Aβ, and (in the context of ischaemia) this cytokine has been shown to decrease CBF by releasing ET [125], although it is unknown whether this decrease is generated by pericytes. Similarly, a mutation in the microglial TREM2 receptor (an AD susceptibility gene) that increases the production of inflammatory mediators also leads to a decrease of CBF [85]. The neuroinflammation occurring in multiple sclerosis can also be associated with hypoperfusion that is correctable by blocking ET_A_ receptors or voltage-gated calcium channels [33, 34].

## The role of upstream arteries and arterioles

Constrictions of rodent cerebral arterioles and middle cerebral artery, resulting in a decrease of cerebral blood flow, have been reported to be evoked by application of exogenous Aβ_1–40_ [130, 169], but interestingly—at least in the APP^NL−G−F^ rodent model of AD—the level of Aβ that occurs in AD is sufficient only to constrict capillaries and not arterioles [133]. Nevertheless, in some AD mice, neurovascular coupling is impaired at the arteriole level [131]. Furthermore, changes in the properties of arteries and arterioles upstream of the brain’s capillary beds, and of the downstream venous system, could contribute to the onset of AD. Possible contributing changes include atherosclerosis [69, 182] leading to partial occlusion of large vessels, an increase in arterial stiffness [69] and hypertension [45, 72] (discussed below) resulting in microvascular damage. It is possible that, rather than directly reducing CBF, these changes may promote Aβ generation or reduce its clearance [45, 69].

## Capillary block by neutrophils and clot formation also reduce CBF in AD

The graded constriction of capillaries by pericytes is predicted to reduce CBF by 50% even in the absence of cells in the blood [133]. In addition, two mechanisms that can produce complete occlusion of vessels have been reported to reduce CBF in AD.

By imaging cell movements in cerebral capillaries, Cruz Hernández et al. [26] observed that in AD (APP/PS1), mice capillaries could become blocked by neutrophils (Fig. 2e). In the AD mice 1.8% of capillaries—predominantly of smaller diameter—became blocked, whereas in wild-type mice only 0.4% of capillaries were blocked. It will be important to reproduce these results in human AD patients. In wild-type mice, capillary block increases with ageing and can lead to vessels being pruned [159]. Remarkably, although modelling suggested that the increased block in AD would lead to a decrease of CBF of less than 5%, applying intraperitoneally a high concentration of an antibody to a neutrophil surface marker (Ly6G) led to a relief of capillary block, an increase of blood flow by 26–32% and improved memory. This is surprising because, at least in conditions of inflammation, antibody to Ly6G promotes neutrophil adhesion and aggregation, coagulation and decreased blood flow [132]. The large effect of the antibody on CBF compared with the modelling predictions for relief of capillary block alone may indicate either that the modelling is over-simplified or that the antibody has effects beyond simply preventing neutrophil blocking of capillaries, perhaps on the effective viscosity of the blood (which leukocytes significantly affect [2, 16]) or on interactions with platelets and endothelial cells [110].

Cortes-Canteli et al. [25] employed long-term anticoagulation with a direct oral anticoagulant, dabigatran, to try to improve outcome in AD mice, based on the observation that excess fibrin is deposited in the AD brain, indicating an excessively prothrombotic environment. Dabigatran preserved CBF and reduced cognitive decline in AD mice (Fig. 2f). While a 15% decrease in CBF was seen at 40 weeks of age in AD mice (a smaller decrease than occurs in affected regions in human AD, possibly because cortical CBF was assessed by measuring it relative to thalamic CBF, which may itself be decreased [11]), after anticoagulation treatment from 2 months of age the CBF was raised above normal by 13%. Interestingly in humans receiving oral anticoagulants, the risk of dementia is reduced by 29% [44].

Given the profound constriction of cerebral capillaries at pericyte somata that is observed in biopsies from human patients developing AD, from a diameter of ~ 5 to ~ 2.8 μm [133], it is attractive to hypothesize that both the block of capillaries by neutrophils and the formation of clots that reduce CBF are a consequence of the reduced diameter of capillaries near pericyte somata. Neutrophils are larger and less distensible than red blood cells and pass through capillaries more slowly [16, 37], and so may tend to become lodged at the smallest diameter parts of capillaries. Similarly, although Cortes-Canteli et al. [25] did not image the vasculature to define which vessels exhibited coagulation, the decreased flow expected through pericyte-constricted capillaries would tend to promote clotting, suggesting that thrombi forming in the smallest vessels may contribute to the reduction of CBF occurring.

## Capillary constriction and reduced CBF accelerate AD onset

The capillary constriction seen in AD leads to the neural tissue becoming hypoxic [133], which presumably contributes to the decrease in glucose metabolism observed in AD (see above). Importantly, ischaemia and hypoxia have been shown to upregulate the enzyme (BACE1) responsible for generating Aβ [168, 197], as schematised in Fig. 3. This leads to more Aβ production [168, 197], which is expected to promote neurodegeneration and cognitive decline in accordance with the amyloid hypothesis, and indeed this was found [168, 197]. While these mechanistic studies were all in animals or on cell lines expressing human BACE1, the level of BACE1 and its enzymatic activity are increased in humans suffering from AD [78], as expected from the fact that the capillary constriction in humans developing AD is sufficient to reduce cerebral blood flow by up to 50% [133] and the animal work cited above showing that ischaemia and hypoxia upregulate BACE1. Furthermore, an upregulation of BACE1 has been found to exist in mild cognitive impairment patients, and correlates with Aβ plaque number and cognitive decline [23]. The upregulation of BACE1 by ischaemia and hypoxia occurs as a result of caspase-3 both increasing BACE1 mRNA level and cleaving GGA3, an adaptor protein involved in BACE1 trafficking, to decrease BACE1 degradation [171, 184, 194], and has two conceptual implications.

Firstly, BACE1 upregulation implies that low blood flow or hypoxia—caused by a purely vascular defect, brain injury, sleep apnoea or genetic predisposition—could initiate the production of Aβ. Indeed, bilateral occlusion of the carotid arteries leads to Aβ production and a fall of metabolism in the amygdala, entorhinal cortex and hippocampus [140]. This could explain why subjects with sleep apnoea, or head injury that decreases CBF [155, 178], are more likely to develop AD [95, 189]. Similarly, hypertension leads to a 45% decrease of CBF in selected brain regions [27, 72, 124], and the resulting upregulation of BACE1 may contribute to Aβ accumulation and the increased likelihood of suffering from AD that is associated with hypertension [72]. For the severe ischaemia produced by stroke, however, it is debated [46, 154] whether this evokes Aβ deposition that contributes to the increased incidence of dementia that occurs post-stroke [117]. Some genetic variants may act by reducing CBF. The ApoE4 variant of ApoE is the main susceptibility gene for AD, and has important vascular effects. Expression of ApoE4 leads to a lower CBF even in cognitively normal subjects [111], which will tend to upregulate BACE1 and increase Aβ production (see above). It also promotes accelerated loss of pericytes and consequent breakdown of the blood–brain barrier, which correlate with cognitive decline [119]. Since experimentally reducing CBF also leads to pericyte loss [41, 56, 97] and hence BBB breakdown [4, 13, 97, 118], it is unclear whether the primary effect of ApoE4 on pericytes is to make them constrict capillaries (ApoE4 is known to affect the cytoskeleton and so may affect contractility [22]) with the resulting decrease in CBF causing pericyte loss and subsequent BBB breakdown, or whether the primary effect is the loss of pericytes which somehow causes a decrease of CBF.

Secondly, once Aβ production (or an imbalance between production and removal by various mechanisms described below) has been initiated, the resulting constriction of capillaries by pericytes that it initiates (see above) will reduce CBF, causing an upregulation of BACE1 and production of more Aβ (Fig. 3). This positive feedback loop will amplify the production of Aβ, over an as yet unknown time course, resulting in a further imbalance between Aβ production and removal.

## Capillary constriction as a link between Aβ and tau phosphorylation

Downstream of Aβ production, an important driver of cognitive decline is tau hyperphosphorylation [57, 62, 91], which leads to tau dissociating from microtubules, aggregating and localising more in dendrites (Fig. 3). Importantly, ischaemia (or hypoxia), which is evoked by the pericyte-mediated capillary constriction that Aβ evokes [133], is known to trigger tau phosphorylation [140, 145, 147]. This is unlikely to reflect solely the increase in Aβ level evoked by ischaemia/hypoxia discussed above, because tau phosphorylation occurs in hypertensive rats (which are ischaemic and hypoxic) even without Aβ pathology [147] and is evoked by unilateral carotid artery occlusion in AD mice without a rise in Aβ_1–42_ level [145].

Major enzymes phosphorylating tau at AD-related sites include Cdk5 (cyclin-dependent kinase 5) and GSK3 (glycogen synthase kinase 3) [52, 91, 98]. For the following reasons, these may be activated by capillary constriction which evokes ischaemia/hypoxia, and thus inhibits Ca^2+^ pumping out of cells and raises [Ca^2+^]_i_. Cdk5 is activated when a raised [Ca^2+^]_i_ activates calpain to cleave Cdk5′s regulatory subunit p35 [98, 158]. GSK3 is activated by prolonged hypoxia via a decrease in activity of the phosphatidylinositol 3-kinase/Akt pathway [122, 191] and on a shorter time scale by an imipramine-sensitive mechanism [149].

Thus, the Aβ-evoked reduction of CBF, produced by pericyte-mediated capillary constriction in AD, could provide an important link between the rise of extracellular Aβ concentration and the hyperphosphorylation that leads to tau relocating to dendrites and impairing synaptic function (Fig. 3). Consequently, cognitive decline is likely to involve a reduction of CBF, whether the cognitive decline is produced ultimately by Aβ or by tau hyperphosphorylation.

## Effect of reduced blood flow on Aβ clearance and blood–brain barrier (BBB) in AD

The CNS is presumably exposed mainly to Aβ generated within the CNS, rather than Aβ generated peripherally and entering across the BBB (although Aβ transfer in this direction is possible via the receptor for advanced glycation end products (RAGE) [32]), Consequently, the rise of CNS Aβ concentration that occurs in AD depends not only on the rate of Aβ production, but also on the rate at which it is enzymatically degraded within and removed from the CNS [108]. This raises the question of how Aβ clearance will be affected by the up to 50% reductions of CBF that occur in affected areas [5].

Four major clearance routes for Aβ from the CNS have been proposed: via efflux across endothelial cells into the blood; via bulk extracellular flow into the CSF and lymphatic vessels; via movement through the perivascular spaces of either penetrating arterioles or alternatively venules (promoted by cardiac cycle driven pulsation of arterioles and, in the case of exit along venules, also water flow through astrocytes termed the glymphatic system: see below); and via phagocytosis and subsequent degradation by microglia, astrocytes and other cells. Injections of radioactive Aβ into the brain parenchyma have been used to try to quantify the relative importance of these removal mechanisms [160]. Five hours after injecting Aβ_1–40_, 84.5% of it had been cleared from the CNS and 15.5% was retained. The retained material might include Aβ in the interstitial space and Aβ (or breakdown products) sequestered in microglia, astrocytes and other cells. Of the removed Aβ, 12.7% (i.e. 10.7% of the total injected) was removed by a process that also occurred for the inert tracer inulin, which may include all mechanisms driven by interstitial fluid flow. The remaining 87.3% of removed Aβ was assumed to have exited the BBB across the endothelial cell layer of capillaries. Similar experiments showed that (at 30 min after tracer injection) 30% more Aβ_1–42_ than Aβ_1–40_ was retained in the brain and correspondingly less was cleared across the BBB [196]. Clearance across the BBB involved PICALM (phosphatidylinositol-binding clathrin assembly protein [196]), which is expressed in vascular endothelial cells [190], and LRP1 (low density lipoprotein receptor-related protein 1; but see [75]), which is expressed in perivascular astrocytes and pericytes and to a small extent in capillary endothelial cells (as well as neurons, microglia and oligodendrocyte precursor cells [190]). A major role for endothelial cell LRP1 in mediating Aβ export is shown by knock-out work [167], but astrocyte and neuronal LRP1 may also be involved [79, 96]. There is evidence for association of PICALM and LRP1 gene variants with human AD risk (reviewed by [161] and [196]).

The decrease of CBF that occurs early in preclinical AD could decrease Aβ removal across endothelial cells, thus potentiating Aβ accumulation, by decreasing the level of proteins that mediate the removal. For example, ischaemia will raise [Ca^2+^]_i_ which can result in calpain cleaving PICALM [150], and indeed PICALM levels are lower in human AD, correlating both with an increased Aβ level and with cognitive decline as assessed with the Mini Mental State Exam [196]. Similarly, ischaemia leads to the endopeptidase furin cleaving LRP1 [185]. Additionally, a slowing of capillary blood flow could in principle allow Aβ that has exited into the blood to re-enter the brain parenchyma by RAGE-mediated entry across endothelial cells [32], thus again slowing net removal of Aβ.

The CBF decrease in AD is also expected to alter Aβ removal by the other, apparently quantitatively less important [66, 160], mechanisms mentioned above. Pulsation of penetrating arterioles during the cardiac cycle or spontaneous vasomotion has been postulated to power the removal of Aβ (in a retrograde direction with respect to CBF) in the perivascular spaces of penetrating arterioles [36, 156]. Arteriole pulsation is also presumed to promote water flow along the paravascular spaces of arterioles and through both aquaporin-4-expressing glial cells and the extracellular space of the brain [73, 74]. This flow may reach: (i) venules, where it helps to remove Aβ in the perivascular spaces of venules (in the same direction as CBF [73]), and (ii) the CSF and lymphatic vessels [6, 100, 103, 137]. A detailed analysis of these proposals has been provided [66, 164]. In AD, when CBF decreases, decreased pulsatility of the middle cerebral artery has been reported [134] and so, if this extends to penetrating arterioles, less Aβ removal by pulsation-driven mechanisms would be expected. Indeed, removal of Aβ by the CSF, lymphatic and glymphatic systems decreases in AD [88, 142], possibly with contributing factors including increased stiffening of the arterioles with age [179] and ischaemia-induced changes of other key components such as decreased lymphatic function and aquaporin 4 localisation away from astrocyte endfeet abutting blood vessels [28, 88, 186].

The CBF decrease induced by capillary constriction in AD may also alter microglial and astrocyte removal and degradation of Aβ. Ischaemia followed by reperfusion (which may mimic the prolonged decrease of CBF occurring in AD) decreases microglial ramification [106, 121], which could decrease Aβ removal by these cells as surveillance of the brain parenchyma will be reduced [104]. On the other hand, ischaemia upregulates expression of triggering receptor expressed on myeloid cells-2 (TREM2), which is a key molecule by which microglia recognise Aβ and remove it [138, 195], as well as other phagocytosis-related genes [192], suggesting an enhanced ability to remove Aβ by microglia. Similarly ischaemia upregulates ABCA1, MEGF10 and GULP1, which are components of an astrocytic phagocytosis pathway [120], suggesting that the CBF reduction occurring in AD may also enhance Aβ removal by astrocytes [www.biorxiv.org/content/10.1101/2020.03.29.002857v1].

Although this review focuses on the effects of the reduction of CBF that is induced by pericyte-mediated capillary constriction in AD, pericytes themselves are very sensitive to ischaemia [41, 56]. In AD the reduction of CBF, together with intracellular accumulation of Aβ in pericytes [181], may eventually lead to pericyte death [41, 56], which will lead to a loss of BBB function [4, 13, 118, 126] that promotes neurodegeneration [153].

## The role of white matter CBF changes in the onset of AD

Although most attention in the AD field focuses on changes in the grey matter, the Aβ level also increases in the white matter in AD [24], and the CBF decrease early in AD occurs in the white matter as well as the grey matter [80]. Consequently, the CBF decrease might exert some of its effects by generating white matter dysfunction, such as slower action potential propagation. White matter tissue is lost before grey matter tissue in AD [30], and early in AD white matter abnormalities defined by MRI correlate both with cognitive decline and with reduced CBF in the deep and circumventricular white matter [18, 77, 93]. Surprisingly, however, white matter capillary diameter has been reported to increase in AD [61]. These results suggest that it will be important to determine whether, in preclinical human AD, capillary constriction by pericytes occurs in the white matter, as in the grey matter [133], or whether CBF decreases as a result of upstream vessel constriction in the grey matter [101] (possibly with dilation of white matter capillaries as an adaptive response) or for some other reason, and to establish precisely which downstream mechanisms (such as myelin loss [116]) lead to white matter dysfunction early in AD.

## Implications for therapeutic approaches to Alzheimer’s disease

**Fig. 4:**
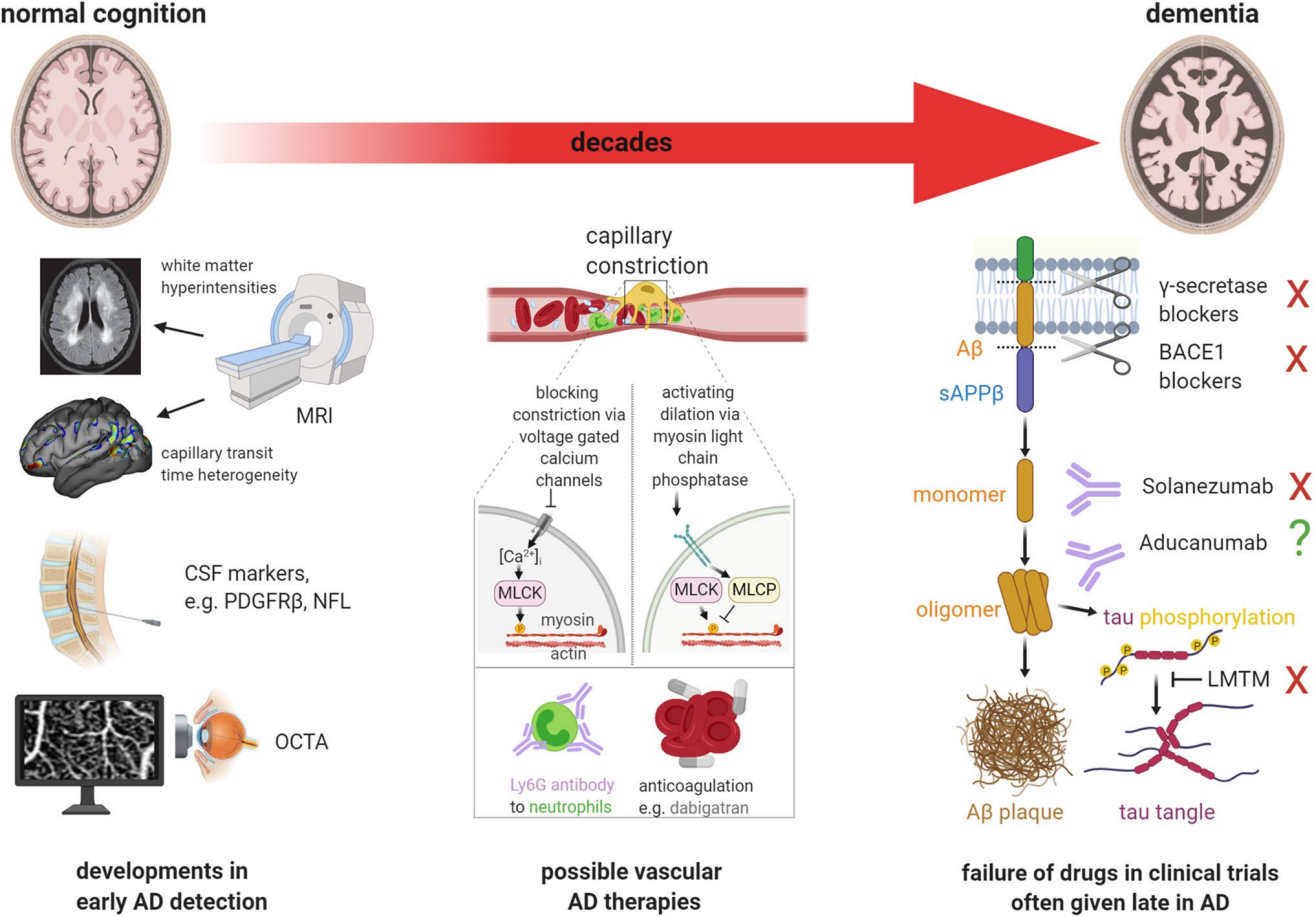
Interventions to diagnose and reduce cognitive decline at different stages of the transition from normal cognition to dementia in AD. Right third of figure: most clinical trials are initiated at relatively late stages of the disease, when cognitive decline is already apparent, and irreversible synapse or neuron loss may have taken place. This may explain why drugs that block the γ or β secretases, antibodies to different forms of Aβ, and a drug that blocks tau aggregation (LMTM) have all failed (red crosses) to stop cognitive decline in AD. Left third of figure: emerging diagnostic approaches for early detection of AD include MRI assessment of white matter hyperintensities (image from Fig. 1B of [93], reproduced courtesy of Dove Medical Press) and capillary transit time heterogeneity (from Fig. 5E of [128], reproduced courtesy of John Wiley & Sons), assessment of biomarkers in the CSF such as PDGFRβ and neurofilament light chain (NFL), and non-invasive capillary imaging in the retina using (e.g.) optical coherence tomography angiography (OCTA). Middle third of figure: potential therapies to prevent or reverse the CBF decrease arising when Ca^2+^ activates myosin light chain kinase (MLCK) to evoke pericyte-mediated capillary constriction. These include blocking pericyte voltage-gated calcium channels to block Ca^2+^-evoked constriction, raising pericyte cGMP level (by activating guanylate cyclase receptors, blue membrane protein) to stimulate myosin light chain phosphate (MLCP) and thus evoke dilation, disrupting neutrophil surface interactions with endothelial cells or other cells using antibodies (if this approach can be used without inducing neutropenia), or blocking thrombus formation with dabigatran [25, 26, 133]

The discoveries that the decrease of CBF in AD occurs early in the disease [76], and is caused by impaired capillary regulation of CBF [26, 54, 128, 133], are consistent with the proposal that impaired capillary blood flow contributes to the onset of AD [31] made soon after the amyloid hypothesis of AD was proposed [59]. These data, including the demonstration that Aβ itself can trigger pericyte-mediated capillary constriction [133], reconcile genetic evidence for the involvement of Aβ in AD with the fact that the first change seen in AD is a decrease of cerebral blood flow [76], and open up new potential therapeutic approaches for this disease. Conceivably, maintaining CBF may prevent cognitive decline if interventions are made early enough to avoid neuronal and glial damage. Just as the risk of stroke is now reduced by giving blood pressure lowering drugs prophylactically, we expect the long-term future of AD therapy to involve—at least partly—prophylactic agents that prevent pericyte-mediated capillary constriction, and thus prevent both direct effects of CBF decreases and the amplification of Aβ production and tau phosphorylation that a fall in CBF generates. Below, we consider approaches to achieving this and possible biomarkers to use to decide when prophylaxis should be initiated (Fig. 4).

### Preventing pericyte-mediated capillary constriction

The constriction of capillaries by pericytes may be mediated by Aβ evoking the generation of ROS that trigger the release of endothelin-1 (ET), which activates [Ca^2+^]_i_-elevating contractile ET_A_ receptors on pericytes [133]. Indeed, in short-term experiments, blocking ROS production and ET_A_ receptors prevented development of further Aβ-evoked constriction [133]. However, long-term block of ROS generation is undesirable because ROS are used for signalling in many contexts, as well as for immune defence mechanisms. Furthermore, ET_A_ receptor activation is difficult to reverse with blockers [64], and although there is a BBB-permeable ET_A_ receptor blocker licenced for clinical use (clazosentan for sub-arachnoid haemorrhage), side effects make this drug unsuitable for long-term administration [175].

A better approach to preventing capillary constriction may therefore be to inhibit the contractile pathways downstream of ET_A_ receptors by blocking the release of Ca^2+^ from internal stores and increasing the activity of myosin light chain phosphatase to activate relaxation of the contractile filaments. These twin aims can be achieved by using an agonist of guanylate cyclase receptors, such as C-type natriuretic peptide (CNP, [166]). Indeed, CNP rapidly reverses Aβ-evoked constriction of capillaries in brain tissue [133]. An alternative approach is to relax pericytes by inhibiting their voltage-gated Ca^2+^ channels (VGCCs). Interestingly, comparing different classes of drugs used to reduce hypertension, it has been claimed that only VGCC blockers slow the progression to dementia in AD ( [102], see also [183]), although not all VGCC blockers used for hypertension cross the BBB well and there are numerous mechanisms by which they may slow cognitive decline [90]. One BBB-permeable VGCC inhibitor, nilvadipine, has been shown to restore the CBF of AD mice to normal levels [139]. In human AD, although nilvadipine lowers peripheral blood pressure, it increases CBF in the hippocampus [29], presumably by relaxing pericytes, and shows some slowing of cognitive decline in very mild AD patients [1]. Devising ways of targeting VGCC blockers specifically to CNS pericytes might enhance the efficacy of this approach. Firstly, it would be desirable to avoid inhibiting VGCCs in neurons, which might be achievable by using bivalent drugs that also bind to proteins expressed relatively specifically by pericytes, such as PDGFRβ. Secondly, if it were possible to avoid inhibiting VGCCs in pericytes and smooth muscle cells around peripheral blood vessels, this would probably avoid the decrease in blood pressure that stems from relaxing the vasculature all over the body.

### Preventing neutrophils occluding capillaries

As noted above, Cruz Hernández et al. [26] showed that, in AD mice, brief application of an antibody to the Ly6G protein on neutrophils increased CBF by 30% and improved memory, although in aged AD mice the cognitive effect was absent, presumably because too much synaptic damage had occurred by that stage [15]. The improvement of cognition in parallel with the increase in CBF in younger AD mice strongly supports the concept of devising interventions to preserve CBF in AD. However, prolonged application of antibody to Ly6G leads to very significant neutropenia (a depletion of neutrophils) within hours [26], which will engender a heightened risk of infection, and thus is not suitable as a long-term therapy (this approach has not yet been used in humans). Thus, further research is needed to devise an agent which generates the blood flow increasing effect of Ly6G (which may be via more than one mechanism: see above) without causing neutropenia. As with the approach of targeting pericyte-mediated capillary constriction discussed above, it will also be necessary to consider the overall effect on blood pressure caused by a manipulation that decreases vascular resistance throughout the body.

### Use of anticlotting agents

The prolonged use of anticoagulants to improve cerebral blood flow and outcome in patients liable to developing AD [25] might lead to an increased risk of intracranial haemorrhage. AD often coexists with cerebral amyloid angiopathy (CAA), for which asymptomatic micro-bleeds, bleeding into the cortical sulci and large symptomatic lobar cerebral haemorrhages can be complications. These are thought to be due to a breakdown in microvasculature integrity as Aβ accumulates along vessel walls and injures them [50]. Criteria exist for diagnosing CAA [51], and detection of intracerebral haemorrhage (including micro-bleeds) has been greatly enhanced by T2*-weighted MRI imaging sequences with a high sensitivity for bleeding [50]. However, further research is required to determine whether there are specific CAA-related biomarkers that would help clinicians to recognise and exclude those patients who would be put at an unacceptable risk of serious intracerebral haemorrhage from anticoagulation, before it could be adopted as a widespread prophylactic treatment for AD.

### Relevance of these approaches to other neurodegenerative disorders

The Aβ-evoked constriction of capillaries by pericytes may involve ROS generation that evokes the release of endothelin-1 [133]. ROS generation also occurs when α-synuclein accumulates in Parkinson’s disease (PD) and Lewy body dementia (LBD) [12, 14], and may evoke ET release and constrict capillaries as for AD. Indeed, PD and LBD are associated with decreased cerebral blood flow [42, 170]. Accordingly, the therapeutic approaches outlined above may also be relevant to these disorders.

### Choice of biomarker for initiating treatment

To date, candidate treatments for AD have almost certainly been initiated too late, after irreversible damage to the brain has occurred, as a result of making treatment decisions based on significant observable cognitive decline. If we are to move towards more preventative treatments, they will need to be started as soon as the earliest changes occur in the disease, raising the question of what biomarkers to use to trigger treatment. Assuming that pericyte-mediated capillary constriction is indeed a very early event in the onset of AD (see Fig. 3) as suggested by Iturria-Medina et al. [76] and Nortley et al. [133], it will become essential to develop non-invasive tests to detect the onset of capillary constriction near pericytes. Markers of cell damage, such as CSF levels of neurofilament light chain which may indicate damage to white matter axons [35] or PDGFRβ for pericytes [118], while useful for assessing the extent of neurologically relevant damage, may only be detectable too late for initiating a preventative drug strategy.

Techniques that look directly at deleterious decreases of CBF (which may follow a period of adaptive hyperperfusion in some brain regions [40, 53, 180]), and its capillary control, may therefore be preferable. In human patients, MRI can be used to measure CBF. Dynamic susceptibility contrast MRI with an injected tracer has been used to quantify changes of blood capillary transit time (and its heterogeneity) in early AD [38, 128], which we argue above probably reflect pericyte-mediated constriction of capillaries. If these measurements could be performed using non-invasive (i.e. without an injected tracer) arterial spin label MRI, then it would provide a method to assess changes in how pericytes control blood flow in different capillaries. An alternative, more direct, observation of pericyte-mediated capillary constriction may be possible by imaging retinal capillaries through the intact cornea, using optical coherence tomography angiography (OCTA), which has been used to detect decreases in neurovascular coupling at the arterial level [146]. OCTA could perhaps thus provide a screening method for detecting pericyte malfunction early in preclinical AD. Aβ plaques are reported to be deposited in the retina before being deposited in the brain [86]. Thus, pericyte-mediated capillary constriction evoked by Aβ oligomers should also be detectable early on as a focal reduction of capillary diameter around pericytes (cf [133]), although this reduction is likely to be close to the limit of resolution of the OCTA technique and this approach would require validation with post-mortem immunohistochemistry.

## Conclusions

With the discoveries that a decrease of cerebral blood flow is the earliest change to occur in AD [76], that this is generated at the capillary level [26, 38, 133] and that changes in capillary control of CBF correlate with cognitive decline [128], it is becoming impossible to ignore the vascular contribution to Alzheimer’s disease. The reduction of CBF produced by pericytes constricting capillaries, along with ensuing decreases in CBF as a result of capillary occlusion by neutrophils and thrombi, is an important dysfunction in AD that potentially opens up new therapeutic approaches and new screening possibilities. Initial evidence indicates that reversing this reduction of CBF can restore cognitive function, provided that damage to synapses, neurons and circuits has not advanced significantly. Consequently, in addition to manipulation of other effects of Aβ and tau, devising screening tests to allow therapeutic intervention to maintain CBF should be a key aim for the future treatment of AD.
